# Direct Observation of the Dynamics of Single-Cell Metabolic Activity during Microbial Diauxic Growth

**DOI:** 10.1128/mBio.01519-19

**Published:** 2020-03-03

**Authors:** H. L. O. McClelland, C. Jones, L. M. Chubiz, D. A. Fike, A. S. Bradley

**Affiliations:** a Department of Earth and Planetary Sciences, Washington University, St. Louis, Missouri, USA; b Department of Earth and Planetary Sciences, Weizmann Institute of Science, Rehovot, Israel; c School of Earth Sciences, The University of Melbourne, Parkville, Victoria, Australia; d Department of Biology, University of Missouri, St. Louis, Missouri, USA; e Division of Biology and Biomedical Sciences, Washington University, St. Louis, Missouri, USA; California Institute of Technology

**Keywords:** diauxic growth, methylotrophy, persister cells, SIMS, carbon metabolism, single cell

## Abstract

Population-level analyses are rapidly becoming inadequate to answer many of biomedical science and microbial ecology’s most pressing questions. The role of microbial populations within ecosystems and the evolutionary selective pressure on individuals depend fundamentally on the metabolic activity of single cells. Yet, many existing single-cell technologies provide only indirect evidence of metabolic specialization because they rely on correlations between transcription and phenotype established at the level of the population to infer activity. In this study, we take a top-down approach using isotope labels and secondary ion mass spectrometry to track the uptake of carbon and nitrogen atoms from different sources into biomass and directly observe dynamic changes in anabolic specialization at the level of single cells. We investigate the classic microbiological phenomenon of diauxic growth at the single-cell level in the model methylotroph Methylobacterium extorquens. In nature, this organism inhabits the phyllosphere, where it experiences diurnal changes in the available carbon substrates, necessitating an overhaul of central carbon metabolism. We show that the population exhibits a unimodal response to the changing availability of viable substrates, a conclusion that supports the canonical model but has thus far been supported by only indirect evidence. We anticipate that the ability to monitor the dynamics of anabolism in individual cells directly will have important applications across the fields of ecology, medicine, and biogeochemistry, especially where regulation downstream of transcription has the potential to manifest as heterogeneity that would be undetectable with other existing single-cell approaches.

## Abstract (precis)

Understanding how genetic information is realized as the behavior of individual cells is a long-term goal of biology but represents a significant technological challenge. In clonal microbial populations, variation in gene regulation is often interpreted as metabolic heterogeneity. This follows the central dogma of biology, in which information flows from DNA to RNA to protein and ultimately manifests as activity. At present, DNA and RNA can be characterized in single cells, but the abundance and activity of proteins cannot. Inferences about metabolic activity usually therefore rely on the assumption that transcription reflects activity. By tracking the atoms from which they build their biomass, we make direct observations of growth rate and substrate specialization in individual cells throughout a period of growth in a changing environment. This approach allows the flow of information from DNA to be constrained from the distal end of the regulatory cascade and will become an essential tool in the rapidly advancing field of single-cell metabolism.

## OBSERVATION

In the 1940s, Jacques Monod coined the term “diauxie” to refer to the biphasic growth that often occurs when an isogenic microbial population sequentially consumes two different carbon substrates when both are available in finite amounts (1). The canonical interpretation of diauxic growth is of a population-wide shift in substrate specialization. This model, coupled with population-level analyses, led to the discovery of gene regulation (2, 3). However, recent advances in single-cell technology have revealed ubiquitous heterogeneity in gene expression even within genetically homogeneous microbial populations (4–6). Consequently, in various model systems, hypotheses challenging the canonical model of diauxic growth, including the formation of metabolically distinct subpopulations and coutilization of the available substrates, have emerged (7–9).

Heterogeneity within isogenic microbial populations has been investigated with single-cell genomics (10), epigenomics (11, 12), and transcriptomics (13–15), and single-cell dynamics is mostly known from fluorescent-reporter assays of promoter activity (8, 16). None of these approaches provide information about metabolic specialization downstream of transcription and are therefore many steps removed from a realized phenotype. Despite intensive efforts in recent years to characterize behavior at the single-cell level (7, 8, 17, 18), direct observations of changes in metabolic activity remain only coarsely resolved for individual cells (19). In this study, we used an array of substrates containing isotope labels, and secondary ion mass spectrometry (SIMS) to observe the dynamics of substrate assimilation in populations of individual cells throughout a period of diauxic growth.

Our approach quantifies the integrated history of metabolic activity in each cell by tracking the fate of carbon and nitrogen isotopes. Similar approaches have previously been used to investigate heterogeneity in clonal populations at steady state (20, 21), but by sequentially sampling the population throughout the experimental period, we were able to observe how the distribution of metabolic activities dynamically evolved during the course of the experiment. With SIMS, we measure the isotopic composition of biomass, so it is essential to design an experiment where metabolic specialization can be inferred from this information without ambiguity. Methylobacterium extorquens AM1 is a well-studied facultative methylotrophic alphaproteobacterium that has well-characterized metabolic pathways (see below) and can grow on a variety of substrates, including some single-carbon (C_1_) substrates, such as methanol (M) (22–26). In our growth experiments, we provided a mixture of methanol, which we enriched with ^13^C, and succinate (S) with a natural abundance of ^13^C, enabling the precise fractional contribution-to-biomass synthesis of each substrate to be calculated from the ^13^C/^12^C ratio of each cell’s biomass. Assimilation of C_1_ compounds in *Methylobacterium* requires an active serine cycle pathway. Assessing C_1_ metabolism in this model system represents an advantage over obligate multicarbon heterotrophy (such as in Escherichia coli), where complications may arise in determining metabolic specialization due to the potential for fermentation to occur. In our experiments, the nitrogen source in the experimental medium was enriched in ^15^N compared to that in the acclimation medium, which allowed the total fraction of biomass along each cell’s ancestral lineage since inoculation to be calculated (see Fig. S1 in the supplemental material).

10.1128/mBio.01519-19.1 FIG S1 Definition of isotope space. The 2-dimensional isotope space used to investigate metabolism in individual cells was defined with calibration cultures, with each culture grown to stationary phase over three transfers and with all four combinations of labeled (*) and unlabeled carbon (M and S) and nitrogen (H) sources, namely, S plus H* (A), M* plus H* (B), S plus H (C), and M* plus H (D). The ancestral culture in all cases was fully unlabeled. The labeled methanol substrate consisted of 99% methanol in natural abundance (1.1% [^13^C]methanol) and 1% methanol of which 99% was [^13^C]methanol. Biomass synthesized when grown purely on methanol had an average isotopic composition of 1.90%, which means that on average ∼82% of the carbon atoms in new biomass came from methanol directly, with ∼18% of carbon atoms coming from CO_2_. In our experiments, we assume that this ratio does not change and that biomass synthesized during growth on methanol in our experiments has an isotopic composition of 1.90% ^13^C. (E) Conceptual figure for interpreting Fig. 2. (i) Isotopic composition at inoculation. (ii) Isotopic composition after growth to saturation on pure H* plus S. (iii) Isotopic composition after growth to saturation on pure H* plus M*. Vectors with arrows represent the trajectories through the isotope space of cells growing on either pure S or pure M*. Growth on a combination of S and M* would result in an intermediate trajectory. Cells should theoretically never be found in the grayed-out region of the plot. (iv) Isotopic composition of a hypothetical cell after a single doubling. A cell could have arrived at this position in isotope space through assimilation first of pure M* and then of pure S (solid path), assimilation of pure S and then of pure M* (fine dashed path), or simultaneous assimilation of both M* and S (coarse dashed path). Download FIG S1, PDF file, 1.0 MB. Copyright © 2020 McClelland et al. 2020 McClelland et al. This content is distributed under the terms of the Creative Commons Attribution 4.0 International license.

**FIG 1:**
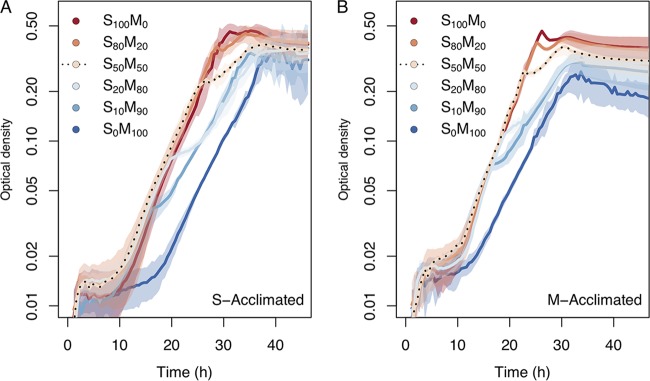
Log-normal diauxic-growth plots for populations of M. extorquens AM1. M. extorquens AM1 was grown in batch culture in liquid media supplemented with a range of concentration ratios of succinate (S) and methanol (M). Prior to inoculation, populations were acclimated to growth in media supplemented with either S (A) or M (B). In experiments with a mixture of S and M, at the level of the population, growth on S was prioritized until S was exhausted, followed by a switch to growth on M. In the key, subscripts refer to percentages of carbon atoms in the medium from each substrate. The optical density reached prior to the diauxic shift (which occurs at the point of S exhaustion) is greater for the population inoculated after being acclimated to methanol due to a short period of initial growth on methanol before switching to succinate. Each solid curve represents the average, and the shaded region represents 1σ across 6 replicate experiments. The black dotted curve highlights the 50% S-50% M (S_50_-M_50_) experiment analyzed at the single-cell level in Fig. 2.

**FIG 2:**
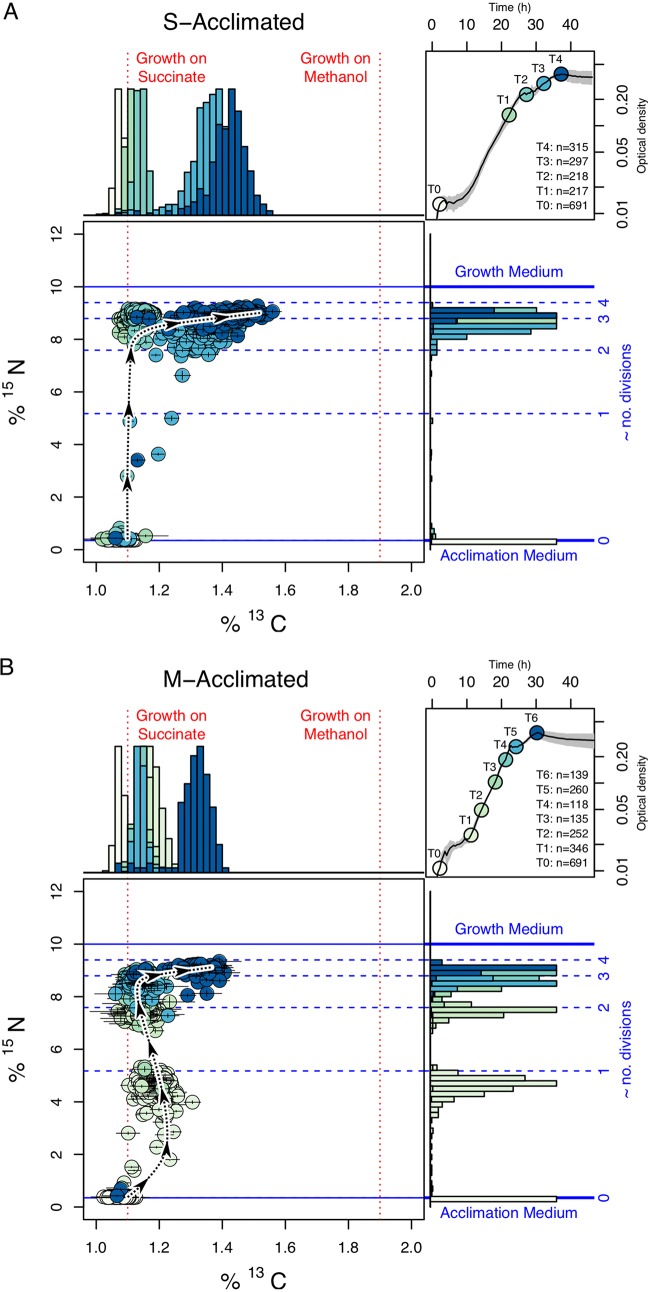
Single-cell metabolism throughout a period of diauxic growth on a 50:50 mixture of isotopically unlabeled succinate, ^13^C-labeled methanol, and a ^15^N-labeled nitrogen source. Each point in isotope space is an individual cell measured with SIMS. Colors correspond to points along a growth curve (top right graphs in each of panels A and B). Acclimation used unlabeled substrates, so in each experiment, inoculated cells inhabit the bottom left corner of the isotope space. Positions on the *y* axis represent the amount of biomass synthesized since inoculation into the experimental medium, and positions on the *x* axis represent the carbon source (see Fig. S1 in the supplemental material for further explanation). In growth curves at the top right of panels A and B, the solid line represents the mean, and the gray-shaded region represents the standard deviation of measured optical densities from six replicate culture experiments measured every 30 min. (A) All cells grew initially on S before transitioning to M upon S exhaustion. Points that plot toward the middle of the isotope space started to grow on M only once S was exhausted (note the color of each point). (B) Most cells initially grow briefly on M before shifting toward S. Once S was depleted, all cells switched to growth on M. Some cells experience a long lag period. The dashed black line highlights the average trajectory of the population through isotope space. T0, time zero; T1, 1-h time point, etc.

When grown on a range of mixtures of succinate and methanol, M. extorquens populations exhibit classic diauxic growth, prioritizing carbon assimilation from S regardless of the acclimation condition (Fig. 1). At the single-cell level, all cells that acclimated to S initially exclusively assimilated carbon from S, followed by a population-wide shift toward M assimilation once S was exhausted (Fig. 2A). Cells acclimated to M initially assimilated M before rapidly switching to assimilate S, and once again assimilating M once S was exhausted (Fig. 2B). Interestingly, we resolve the noninstantaneous nature of the initial shift from growth on M to growth on S in the M-acclimated culture; the curvature of the path through isotope space, as the trajectory changes in the early portion of the experiment (Fig. 2B and Fig. S1E) is diagnostic of the timescales associated with the cellular responses to changing conditions. This observation highlights the “phenotypic inertia” of preexisting proteomic investment in the M metabolic pathways, which must be overcome as cells acclimate to their new environment.

Although we do not see evidence for metabolically distinct subpopulations, there is some heterogeneity in growth. For both the S-acclimated and M-acclimated populations, some cells exhibited lag times of many hours, and a few did not start to grow at all before the last time point of the experiment (Fig. 2). Some with long lag times underwent less than a single doubling on S before all of the S was exhausted, prior to growth on M. Only a small number of cells that had already started growing on S did not make the transition and start assimilating M. The histograms of isotopic compositions at each time point are approximately Gaussian, but with long tails representing few cells with long lag times. The long tails are also evident in the end member experiments that we conducted to define the isotope space (Fig. S1). The width of the histograms in isotope space may arise from differences in protein expression at the level of transcription or translation, microenvironmental differences, metabolic reworking, or the cells at inoculation being in a range of physiological states. Since cellular division is not synchronized, cell sizes at inoculation into the experimental medium are not equal, and even uniform growth rates would result in a spread in isotopic compositions due to the range in maturities of the ancestral cells at time zero.

Our data show that following exhaustion of S, there is a population-wide switch to assimilating M-derived carbon. Methanol metabolism occurs in two steps: (i) oxidation to formate and (ii) assimilation via the serine cycle (24). Every cell that assimilates methanol-derived carbon must have an active serine cycle. However, as this approach tracks the fate of the carbon and nitrogen atoms rather than their route of assimilation, it is possible that some cells lack the ability to oxidize methanol and rather grow on methanol-derived carbon through the scavenging of metabolic intermediates, such as formate, from the medium. Such a scenario is consistent with observations of heterogeneity in the activity of the promoter controlling M oxidation genes (27) and the transient accumulation of the M oxidation product, formate, in the medium during active growth on succinate when methanol is available (24).

We anticipate that this methodology will have important applications in the fields of biology, ecology, and medicine. For example, in the field of infectious diseases, this approach could be used to investigate the dynamics of bacterial “persister” cell formation and regrowth. When a clonal population of pathogenic bacteria is exposed to antibiotic treatment, there is often a small fraction of the population that, despite being genetically identical, is characterized by slower growth and antibiotic tolerance and persists even when the treatment appears successful (28, 29). This latent subpopulation can later lead to regrowth and a relapse in infection (30). The transition into and out of the growth-arrested persister phenotype is poorly understood, but there appear to be stochastic and microenvironmental controls and a number of different mechanisms linking the persister cell phenotype to toxin-antitoxin modules (29, 31). In one proposed mechanism of persister cell formation, nonneutralized toxins lead to inhibition of translation (32) and thus a decoupling between transcription and phenotype. Thus far, single-cell studies directly comparing persister cell metabolism to that of the larger population have relied upon fluorescent-reporter assays to infer transcription and translation (33). As with substrate specialization during diauxic growth, the approach that we have described could be used to directly observe metabolism in persister cells. Indeed, the small but ever-present fraction of the population that exhibited long lag times in our experiments may be analogous to persisters.

From understanding how antibacterial resistance arises in clonal populations of bacteria and the extent and consequences of intercellular metabolic heterogeneity in cancerous tumors, the need for accurate assessment of metabolism in single cells is becoming increasingly important. At the population level, “omics” technologies can be used to comprehensively interrogate cellular metabolism at various points along the path from an organism’s genetic potential to its realized phenotype in a given environment. However, of these rapidly advancing technologies, those that report information about metabolic specialization downstream of transcription are not yet possible for single cells. Moreover, fluorescent-reporter-based analyses require genetic alteration of the subject organism and rely on poorly constrained correlations between transcription and phenotype, which are established at the level of populations. This is where technologies like SIMS provide a novel means of directly observing anabolic specialization in single cells and are suited to analysis of wild-type organisms. SIMS technology in combination with quantitative isotope labeling experiments provides a means of ground-truthing fluorescent-reporter-based analyses, calibrating these relationships at the single-cell level, and testing whether apparent heterogeneity in gene regulation manifests as heterogeneity in activity. In this study, we have been able to observe the final result of this regulatory cascade and directly measured anabolic activity, showing that all cells within an M. extorquens population build biomass from the same extracellular substrate throughout a period of diauxic growth on a mixture of succinate and methanol.

## 

### Strains and media.

Experiments in culture employed the Δ*cel* mutant of the pink-pigmented wild-type AM1 strain of Methylobacterium extorquens (CM2720), which was specifically developed to optimize the analysis of growth *in vivo* (34). Cells were grown in a modified version of Hypho liquid medium (H), consisting of 50 ml phosphate salts solution (25.3 g of K_2_HPO_4_, 22.5 g Na_2_HPO_4_ in 1,000 ml MilliQ water), 50.0 ml sulfate salts solution [5.0 g of (NH_4_)_2_SO_4_, 0.98 g of MgSO_4_ in 1,000 ml MilliQ water], 1.00 ml of 1,000× lanthanide-free trace metal solution (1,000× Z3 trace metal mix, consisting of 0.6 mM Zn, 9.98 mM Ca, 0.51 mM Mn, 8.884 mM Fe, 1 mM Mo, 1.5 mM Cu, 1 mM Co, and 0.169 mM W, with EDTA and stored at pH 2.0), and 899 ml of MilliQ water. Stock solutions were separately autoclaved before being mixed under sterile conditions. Isotopically unlabeled carbon sources, methanol (M) and succinate (S), were prepared as stock solutions in MilliQ to molarities of 2.96 M and 0.74 M, respectively (such that both solutions have the same carbon molarity of 2.96 M), and filter sterilized immediately prior to use. In all growth experiments, substrates were added to the medium to achieve a final total medium carbon molarity of 14.80 mM.

### Methylobacterium metabolism.

In M. extorquens AM1, S enters the tricarboxylic acid (TCA) cycle directly, while growth on M requires the expression of over 100 additional genes (24, 35). M metabolism consists of a dissimilatory phase, where carbon is not incorporated into biomass, followed by an assimilatory phase, where carbon is incorporated into biomass. The dissimilatory phase consists of M oxidation to formaldehyde, catalyzed by methanol dehydrogenase (MeDH) enzymes in the periplasm of the cell, and rapid oxidation to formate via the H_4_MPT pathway in the cytoplasm. Formate can either undergo further dissimilatory oxidation to CO_2_ or be converted to methylene-H_4_F via a sequence of tetrahydrofolate (H_4_F)-dependent enzymes (36, 37). The assimilatory phase begins when methylene-H_4_F enters the serine cycle and is further processed through a partially active TCA cycle and the ethylmalonyl-coenzyme A (CoA) pathway, where it is supplemented with additional carbon in the form of CO_2_ (24). The H_4_F pathway, which is the bridge between the dissimilatory and the subsequent assimilatory phases of M metabolism, is the last part of the complete pathway to be upregulated following a switch from S to M (24). This allows the dissimilatory and assimilatory phases of M metabolism to be decoupled and regulated separately (38), while preventing the catalysis of unwanted side reactions that may compromise the optimization of central carbon metabolism. Although assimilation of M-derived carbon is extremely limited when S is present (38), M. extorquens populations growing on S maintain a low-level capacity to oxidize M to formaldehyde and to formate (24) and further to CO_2_ (38).

### Isotopic compositions of media.

For the isotope labeling experiments, a methanol solution carrying a ^13^C label and modified Hypho medium carrying a ^15^N label (Hypho*) were produced. It was ensured that the only carbon and nitrogen available to the cells was strictly controlled (except where CO_2_ supplements growth on methanol). A 2.96 M stock solution of 100% [^13^C]methanol was mixed with isotopically unlabeled methanol in a 1:99 ratio to produce an isotopically labeled methanol substrate (M*) stock solution. Both isotopically unlabeled M and S substrates consist of 1.07% ^13^C and 98.93% ^12^C, and M* consists of 2.06% ^13^C and 97.94% ^12^C. Similarly, a stock solution of sulfate salts replacing (^14^NH_4_)_2_SO_4_ with 100% (^15^NH_4_)_2_SO_4_ was produced and mixed in a 1:9 ratio with the unlabeled sulfate salts stock solution before being incorporated into the modified Hypho medium previously described to produce H*. Ammonium is the only source of nitrogen provided to the cells; in isotopically unlabeled Hypho, it consists of 0.37% ^15^N and 99.63% ^14^N, and in Hypho*, it consists of 10.33% ^15^N and 89.67% ^14^N.

### Growth experiments.

For each growth experiment, carbon-supplemented medium was inoculated with cells from a parent (acclimation) culture in stationary phase ∼4 h after the maximum optical density at 600 nm (OD_600_) was reached (which peaked at a value of around 0.5) in a 1:99 (vol/vol) ratio. For the acclimation culture, populations were grown to stationary phase three times (three transfers) under identical conditions following inoculation from freezer stocks. Based on a parent culture with an OD_600_ of 0.5 and previous estimates of the relationship between the OD_600_ and cell concentration (37), the initial cell concentration at each transfer was on the order of 2 × 10^6^ cells/ml. A suite of substrate ratio growth experiments and an isotope labeling growth experiment was carried out for each of two acclimation conditions: (i) growth on H plus 100% S and (ii) H plus 100% M.

For the unlabeled carbon substrate ratio growth experiments, cells were grown in 48-well plates and optically analyzed at 30-min intervals with an automated microplate reader (BioTek, Winooski, VT). Six substrate ratio conditions were explored: 100% S; 80% S, 20% M; 50% S, 50% M; 20% S, 80% M; 10% S, 90% M; and 100% M, with six replicates and two blanks (identical media, but they were not inoculated with live cells) for each condition assigned to randomized positions within the plate. There was no systematic influence of well position within the plate on growth, so data from all wells were used.

For the isotope labeling experiments, carbon was supplied in equal amounts in the form of S and M, for both populations acclimated to pure S and to pure M (Fig. 2). The acclimation phase was carried out in unlabeled media, and the experimental phase was carried out in media where the only nitrogen source carried a ^15^N label, the methanol carried a ^13^C label, and the succinate was unlabeled. The nitrogen isotopic composition of each cell represents the total fraction of each cell’s biomass produced since transfer to the experimental medium, and the carbon isotopic composition can be used to quantitatively determine the fraction of carbon atoms in each cell’s biomass that ultimately came from each of the available substrates. Prior to experimental growth, acclimation was undertaken in isotopically unlabeled media before inoculation into new Hypho* medium with 50% S and 50% M* carbon substrates. Cultures were grown under four additional conditions to define the extreme values of the 2-dimensional isotope ratio space that cells must occupy, with growth to stationary phase over three transfers. These conditions were as follows: H plus S, H plus M*, H* plus S, and H* plus M* (Fig. S1).

### Sample preparation for SIMS analysis.

Samples for SIMS analysis were prepared to rigorously avoid contamination of cellular compositions with carbon or nitrogen from an uncontrolled source. For example, cells were not fixed with formaldehyde or stored in 50:50 phosphate-buffered saline (PBS)-ethanol. Aliquots of actively growing cultures were taken at predetermined points along the growth curve and were centrifuged at 4,000 × 9.81 ms^−2^ for 5 min. The supernatant was discarded and the cell pellet gently resuspended in MilliQ water 3 times to remove any traces of salts, extracellular carbon, or nitrogen from the medium or substrates before resuspension in a very small volume of MilliQ. Cells of this bacterial strain are comparatively resistant to osmotic lysis, and most cells survived this process intact. To prepare samples for SIMS analysis, some of the final cell suspension was sprayed in a thin film onto an extremely clean transparent indium- and tin oxide-coated glass disk using an airbrush specifically adapted for the task. The water was evaporated from the glass disk by gently heating it on a hot plate before inspection under an optical microscope. The cells adhered to the slide without the need for additional fixative. Optimal cell densities on the glass disk for SIMS analysis are with cells as close together as possible (i.e., with as many cells as possible in a field of view) but sufficiently separated to allow cells to be separated at the resolution of the SIMS beam. At the resolution used in these experiments, this separation between cells was 2 to 3 cell lengths. The process of layering the cell suspension onto the disk with the airbrush and gently evaporating the residual water was repeated until an optimal cell density was achieved. The optimal area on the disk was then identified and marked on the nonconductive side of the disk.

### SIMS analysis.

Carbon and nitrogen isotopic compositions of individual cells were measured on a secondary ion mass spectrometer (Cameca 7f-GEO). Areas of interest (approximately 100 μm square) were selected via scanning ion imaging, using the criterion of maximizing cell density without compromising individual cell identification (Fig. S2). Scanning ion images of C122−, ^12^C^13^C^–^, ^12^C^14^N^–^, and ^12^C^15^N^–^ were acquired sequentially, using magnet switching and a single electron multiplier detector. (Note that for biological specimens, nitrogen is monitored as CN^–^ [39], and it was then beneficial to monitor carbon as ${\text{C}}_{2}^{-}$, limiting the range of magnet switching to 3 atomic mass units [amu], thereby reducing the likelihood of drift during data acquisition.) The most important isobaric interferences at the nominal masses 25, 26, and 27 amu are ^12^C_2_^1^H^–^, C132−, and ^13^C^14^N^–^, which require respective mass resolving powers of 5,593, 7,152, and 4,272 (M/dm) to achieve mass peak separation. The C132− interference was ignored, because even with maximum label uptake, the probable relative contribution to the ^12^C^14^N^–^ count rate was <0.001. Consequently, the 7f-GEO mass resolving power was set to >5,600. Nominal primary-beam settings were 10 pA Cs^+^, 20 keV net impact energy, and a 1.5-μm diameter beam. A magnetic field settling time of 1.5 s was included prior to each new image acquisition. Cycling continued until all cellular material was sputtered through; there were typically 100 to 400 cycles, totaling between 2.5 and 10 h in duration, depending on the size of the field of view. Around 8 to 10 fields of view were measured for each sample, depending on cell spacing.

10.1128/mBio.01519-19.2 FIG S2 Ion image showing an example of the ideal spacing of cells for SIMS analysis. As in this image, ^12^C^14^N ions were used to select regions of interest (ROIs; cells). The ideal spacing is a tradeoff for getting as large a number of cells as possible in a field of view, with minimal overlapping. Download FIG S2, JPG file, 0.7 MB. Copyright © 2020 McClelland et al. 2020 McClelland et al. This content is distributed under the terms of the Creative Commons Attribution 4.0 International license.

Each region of interest (ROI; i.e., one individual cell) was selected using WinImage software, and all count rates were exported for all ROIs for all cycles. Electron multiplier dead time and quasi-simultaneous arrival corrections (40) were applied as necessary. For each field of view, isotope ratios for all ROIs were plotted against cycle number. Based on numerous “blank” analyses of unlabeled microbes, isotope ratios are not normally distributed around a mean as the cell is sputtered through, being skewed at the onset of sputtering (despite preloading with Cs) and also when the cell is almost consumed. However, excluding these cycles (see Fig. S3 in the supplemental material), histograms of percent deviation from natural abundance of populations of “blank” cells are statistically normal, with typical relative standard deviations of <1% (1σ for >100 cells). Once the cycles for each field of view were chosen, the ratios were averaged across those cycles for each region of interest. Calculated 1σ Poisson uncertainties per cell were consistently below the standard deviation across all cycles for each ROI, as expected for a material that was not perfectly homogeneous. Uncertainties plotted in Fig. 2 represent 2σ of count ratios across all selected cycles for each ROI. Finally, for statistical reasons, the ratio C12C13/C212C13/C12 equals 2, and so the carbon ratio data were scaled accordingly.

Additional data for this article can be found at Mendeley: https://doi.org/10.17632/7f5zbcvpys.1.

10.1128/mBio.01519-19.3 FIG S3 Examples illustrating cycles that were discarded due to effects that occur before stabilization. Shown here are the ion counts per cycle for ^14^N^12^C (top), ^15^N^12^C (middle), and the ratio of these (bottom) for each region of interest through 100 cycles. The thick black line is the average across all ROIs for each cycle, and the dashed blue line is the average across all cycles and ROIs. Cycles in which the cycle average deviated from the global average were discarded. In this example, cycles 1 to 20 were discarded (light gray) and cycles 21 to 100 were kept (dark gray). Download FIG S3, JPG file, 1.1 MB. Copyright © 2020 McClelland et al. 2020 McClelland et al. This content is distributed under the terms of the Creative Commons Attribution 4.0 International license.
