# Supplementary figures and images for: Direct Observation of the Dynamics of Single-Cell Metabolic Activity during Microbial Diauxic Growth
Source: mBio. 2020 Mar 3;11(2):e01519-19. doi: 10.1128/mBio.01519-19 (PMC7064762; doi:10.1128/mBio.01519-19)

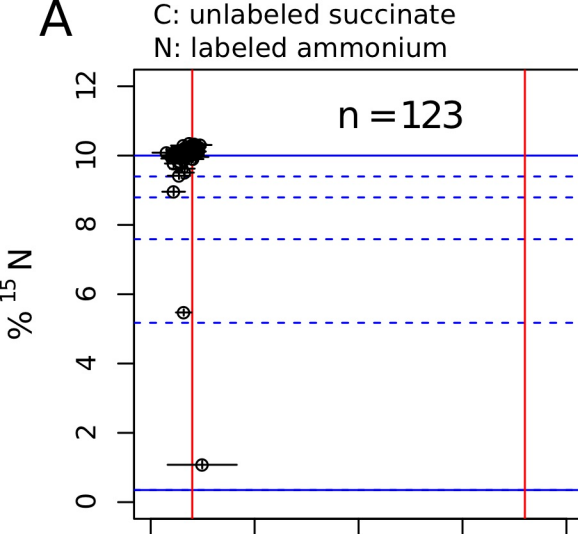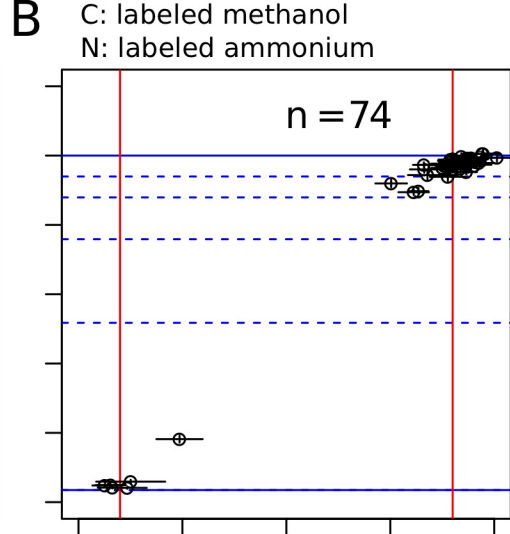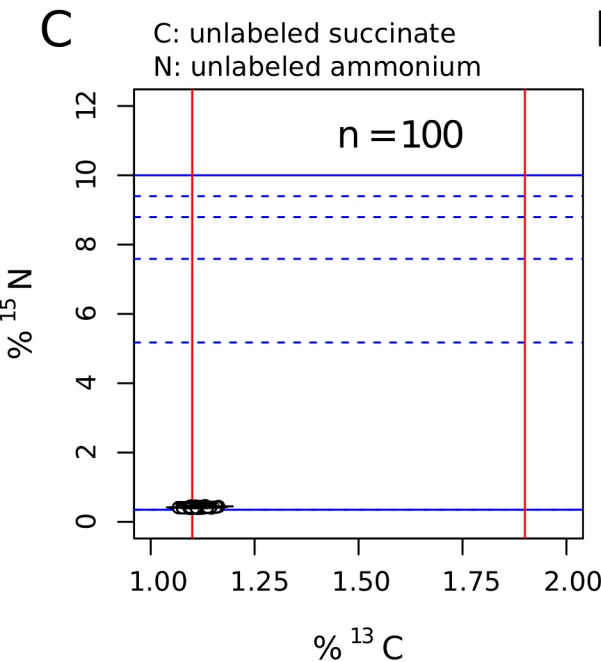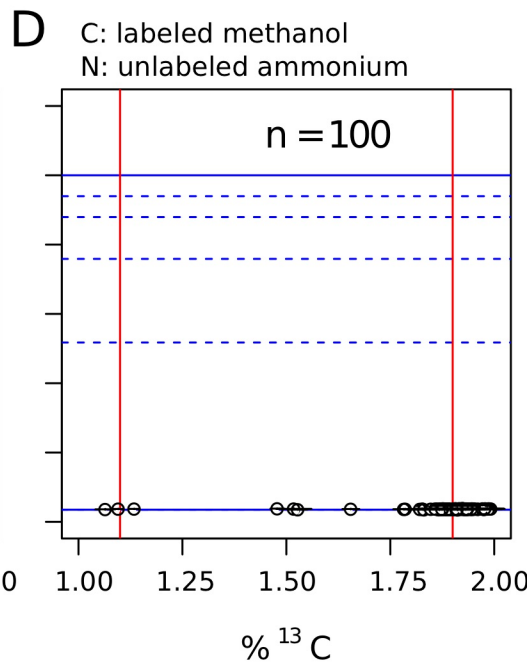

E

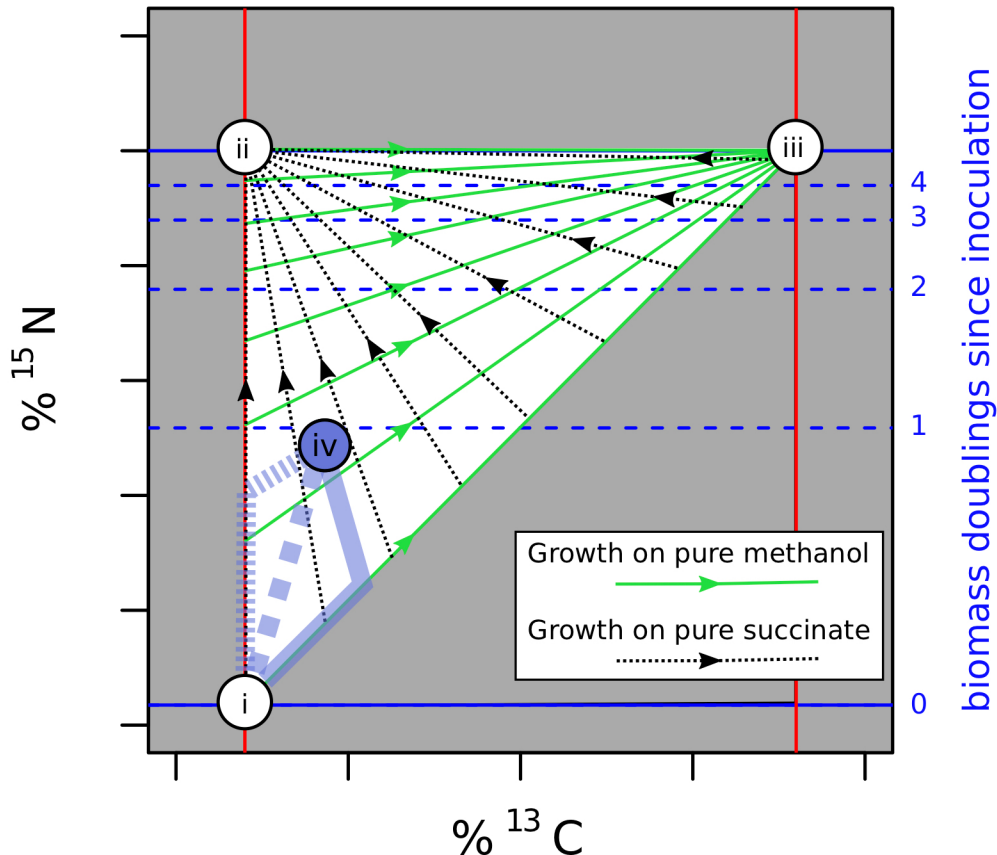

Supplement: FIG S1 [file mBio.01519-19-sf001.pdf]

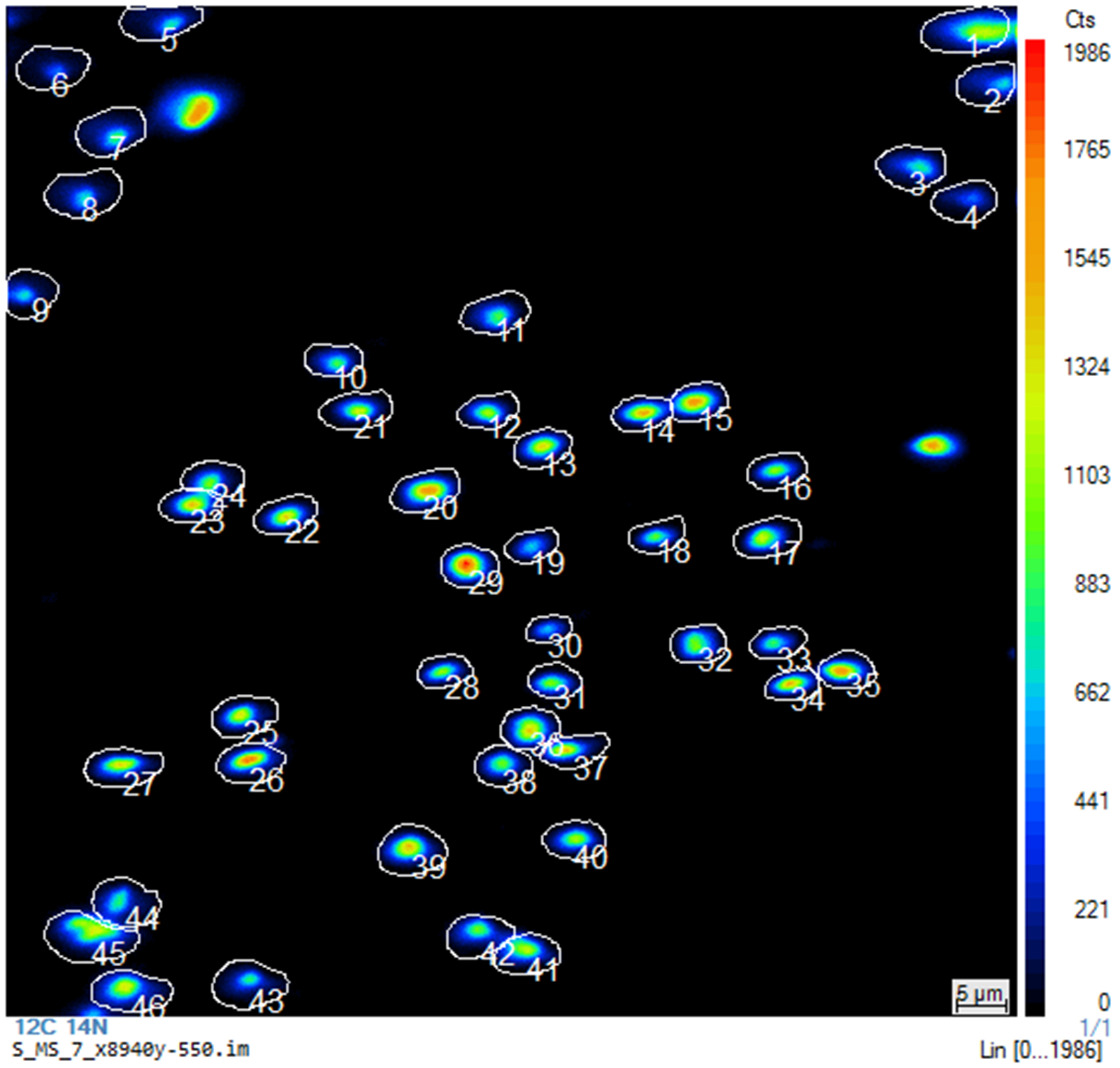

Supplement: FIG S2 [file mBio.01519-19-sf002.jpg]

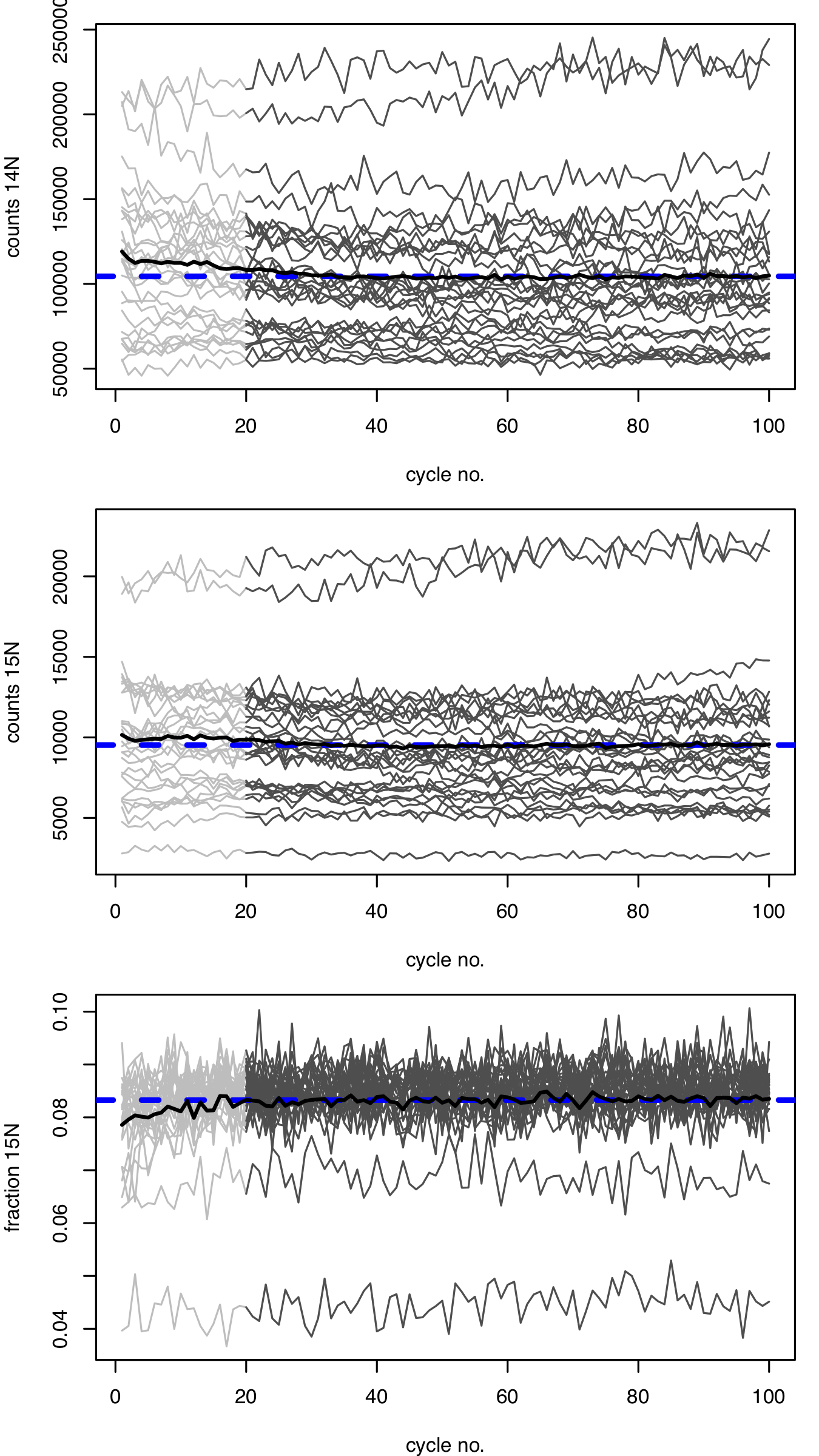

Supplement: FIG S3 [file mBio.01519-19-sf003.jpg]
